# Prominence of the training data preparation in geomagnetic storm prediction using deep neural networks

**DOI:** 10.1038/s41598-022-11721-8

**Published:** 2022-05-10

**Authors:** M. Cristoforetti, R. Battiston, A. Gobbi, R. Iuppa, M. Piersanti

**Affiliations:** 1 FBK, Via Sommarive 18, 38123 Povo, Trento Italy; 2 INFN-TIFPA, V. Sommarive 14, 38123 Povo, Trento Italy; 3 University of Trento, V. Sommarive 14, 38123 Povo, Trento Italy; 4 University of L’Aquila, V. Vetoio, 67100 L’Aquila, Italy

**Keywords:** Magnetospheric physics, Statistics

## Abstract

The direct interaction between large-scale interplanetary disturbances emitted from the Sun and the Earth’s magnetosphere can lead to geomagnetic storms representing the most severe space weather events. In general, the geomagnetic activity is measured by the Dst index. Consequently, its accurate prediction represents one of the main subjects in space weather studies. In this scenario, we try to predict the Dst index during quiet and disturbed geomagnetic conditions using the interplanetary magnetic field and the solar wind parameters. To accomplish this task, we analyzed the response of a newly developed neural network using interplanetary parameters as inputs. We strongly demonstrated that the training procedure strictly changes the capability of giving correct forecasting of stormy and disturbed geomagnetic periods. Indeed, the strategy proposed for creating datasets for training and validation plays a fundamental role in guaranteeing good performances of the proposed neural network architecture.

## Introduction

Today, the possible forecasting of a geomagnetic storm represents the main topic in the space weather context. In fact, many studies have been conducted in order to definitely understand the link among solar processes, interplanetary phenomena and geomagnetic activity (e.g.^1–5^). On the other hand, several works focused their attention on the prediction of the Dst (disturbance storm time) index^6–13^, which measures the dynamic of the symmetric part of the ring current driven by the solar activity^14^. The Dst is an hourly index evaluated using 4 ground-based geomagnetic observatories located at low latitudes (^15,16^ and reference therein). Many statistical and physical models have been developed in order to forecast the Dst index using both interplanetary magnetic field (IMF) and solar wind (SW) parameters data as input (^17^ and reference therein). Simultaneously, other studies derived a function linking SW parameters to magnetospheric energy dynamics (e.g.^18–21^).

On the other hand, many scientists focused on the possibility to predict the Dst index via neural network (e.g.^8,22–27^). Lazzús et al.^23^ was able to efficiently forecast the Dst-index 1–6 h ahead using its past values via artificial neural network (ANN). At the same time, using SW speed, density and the IMF Bz component, Gleisner et al.^28^ forecast the Dst-index 1 h in advance. A better result was obtained by^12^, who used SW plasma density, velocity, flow pressure and IMF components to predict the Dst index 1 h in advance. Finally, Lethy et al.^29^ used IMF Bz, SW electric field, temperature, speed and density to make a prediction of the Dst index via ANN 1–12 h ahead.

## Data and methods

### Dataset

The data used for the present analysis are: the solar wind (SW) plasma parameters; the interplanetary magnetic field (IMF); the Dst index. The entire dataset has been obtained from the National Space Science Data Center of NASA, namely, from the OMNI database^30^. In particular, we used hourly averages of the three components ($B_x$, $B_y$, $B_z$) of the IMF in the GSM (Geocentric Solar Magnetospheric) reference frame (i.e. the x-axis of the GSM coordinate system is defined along the line connecting the center of the Sun to the center of the Earth; the origin is defined at the center of the Earth and is positive towards the Sun; the y-axis is defined as the cross product of the GSM x-axis and the magnetic dipole axis and is positive towards dusk; The z-axis is defined as the cross-product of the x- and y-axes; the magnetic dipole axis lies within the *xz* plane), the SW plasma temperature (*T*), density (*D*), total speed (*V*), pressure (P), and east–west component of the electric field ($E_y$ derived from $B_z$ and $V_x$).

The dataset covers the period January 1990–November 2019, and includes half of the 22nd solar cycle, all of the 23rd, and almost all of the 24th. To produce a robust forecasting of the Dst index, it is crucial to determine how the dataset is split and processed for the training and evaluation of the model. On the other hand, adopting a correct methodology for treating data is crucial to avoid bias especially when both a machine learning approach is used to develop predictive models and the data are time series.

#### Periodicity and arrow of time

If data are periodic, it is safe to train the model considering at least one complete period and test it on different periods. In fact, being the arrow of time fixed and the future unknown, the training operation that make use of points that follow the data used in the test can introduce bias. Therefore, the validation and test data-sets must be constructed by points of the time series that follow what is used for training one. In the present case, since we have only data from two solar cycles, the best option is to use one cycle for training and the other for both validation and test. Anyway, such a choice forces the validation to contain data relative to the first half of a solar cycle with a distribution of Dst values and storms different from the test set. Therefore, in our opinion, the most efficient choice for the validation and test process is to select points randomly for the two datasets.

#### Forecast of rare events (storms)

Training a supervised fashion Deep Learning (DL) model requires both a balanced sampling of data referring to quiet and storm periods, and a proper evaluation of the metrics used to measure the performances. If not, the model will learn to predict only the most frequent case represented in the training set. Moreover, the standard performance metrics, computed on the full validation and test dataset, would produce a the prediction that would be correct most of the time but wrong in most relevant cases.

**Figure 1:**
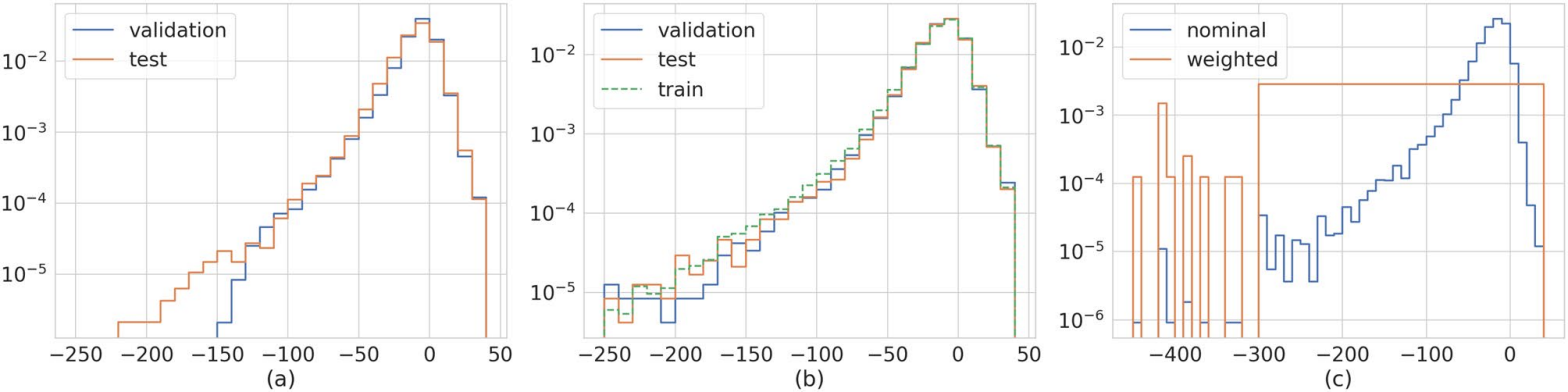
Normalized distributions of Dst in the dataset used for training, validation and test. (**a**) Validation is the first half of the solar cycle period, test the second half. (**b**) Points for validation and test are randomly extracted. Train dataset includes all the available points before 1/1/2009. (**c**) Train dataset without and with re-weighting the low Dst events.

Taking care of these two aspects, we split the dataset using all the data before 1/1/2009 for training, and the remaining part for validation and test. In this way, we have at least one solar cycle for the training and one for the evaluation of the model. As previously said, for the validation and test we can choose dataset subsequent in time (i.e. ordered) or an equal number of points randomly from those available after 1/1/2009. The difference between random and ordered selection are displayed in Fig. 1. In panel a the validation data includes the points in the first half of the cycle while the test is the other half. It is evident that the tail of the two distributions is different: in the validation dataset, events with very low Dst, which are particularly important being connected with storms, are missing. The situation completely changes when the points are picked randomly. In this case, the distributions are quite similar and also similar to the training dataset, representing the best starting point for the development of a data-driven predictive model. The last problem, directly connected to the data distribution, is that there are only few events associated with storms. In the framework used in this paper, where the algorithm learns by looking at the data, if the distribution is highly peaked around some value of the target variable, the algorithm will learn to predict only such values. To avoid this issue, we apply a re-weighting function for the sampling of the data that feed the algorithm’s training. In this way, every value of Dst is almost equally probable. The difference between the *nominal* distribution and the flatten (*weighted*) distribution is presented in Fig. 1c.

The points discussed above limit also the applicability of standard cross-validation methods usually recommended in machine learning applications to test the robustness of the models. While specific schemes of cross-validations have been developed for time series (e.g., the TimeSeriesSplit function available in the Scikit Python library), we prefer not to adopt this type of check because this kind of split increases the size of the training dataset, namely: in the first iterations, there are much fewer storms than in the latest. This automatically will favor the last iterations of the procedure in predicting storms, introducing an indirect bias in the interpretation of the results.

All the features are scaled linearly on a compact range as an additional pre-processing step. The scale is fitted on the training dataset, mapping these min and max values of data in 0.1 and 0.9, respectively. This choice leaves some room to accommodate smaller or larger values than those available in the training dataset that can emerge in future measurements of the variables.

### Model architecture

**Figure 2:**
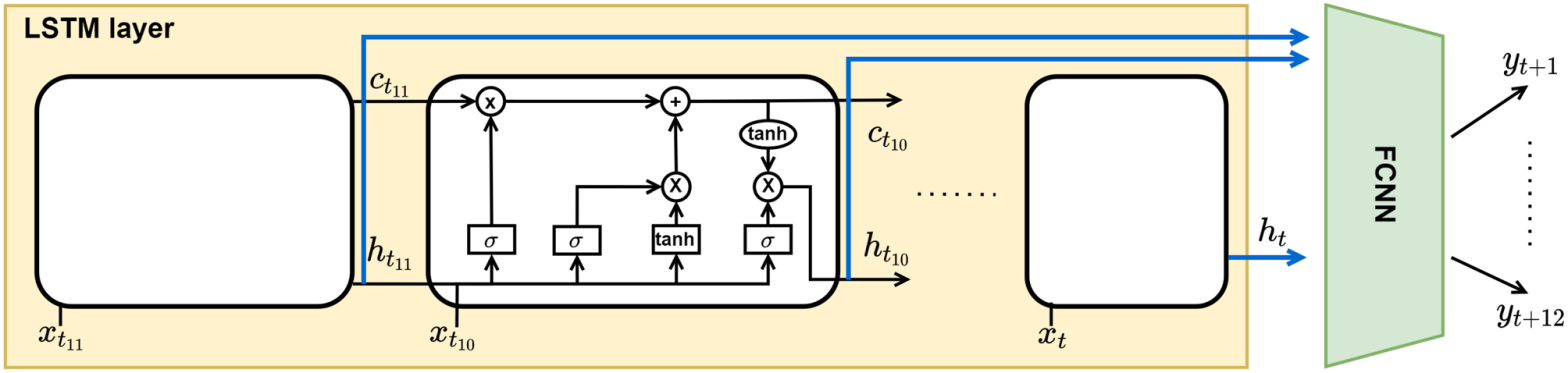
Neural Network architecture used to forecast the Dst index as described in the text. In the LSTM cell, the square blocks are Fully Connected layers with activation function, while the circles are elementwise operations.

The architecture of the Neural Network considered in this study is close to the one used in^26^ where a Long Short-Term Memory (LSTM) module is combined with a Fully Connected Network (FCNN). LSTM is a recurrent layer composed of cells designed to process long time series. The input of the proposed network is time series containing the variables described in “Dataset” for the 12 points in the time window $[t-11, t]$. Each cell of the LSTM layer (Fig. 2) receives in input one element $x_{t_i}$ of this time series together with the outputs of the previous cells: the hidden state, $h_{t_{i-1}}$, and the memory state, $c_{t_{i-1}}$. As schematically depicted in the figure, these three sources of information are processed through fully connected layers and element-wise operations, all internal to the cells. In standard application of LSTM, the hidden state from the last cell represents the network’s prediction, and the hidden states of all the other cells are not considered. In our approach, we collect and concatenate all the hidden states $[h_{t-11}, h_t]$ in a multidimensional vector. This vector is then fed as input of a fully connected module. The output of this FCNN is the forecast of the Dst index for the hours $[t+1, t+12]$.

In optimizing DL networks, two types of parameters need to be fixed: the layers’ weights and the hyper-parameters specifying the architecture. During training, the back-propagation procedure takes care of the former, which can be millions or even billions (in our case 25,244). The others, typically limited in number (in our case 7), are usually determined manually by testing different solutions and considering only the training and validation dataset in the evaluation to avoid bias.

We found that better predictions are obtained using the following values for the hyper parameters:

- LSTM, number of hidden layers: 2,
- LSTM, size of the hidden layers: 8,
- FCNN, number of layers: 4,
- FCNN, number of output features for each layer: 96, 96, 48, 12.

Batch normalization is applied to the input vector of the FCNN, ReLU activation function, and a dropout layer with a drop factor of 0.2 follows every fully connected layer except the last one.

The loss function minimized during the training of the network is the Mean Absolute Error (MAE) function

1 $$\begin{aligned} {\text {MAE}} = \frac{1}{N}\sum _{i=1}^{N}\left| y_{pred} - y_{true}\right| _i \end{aligned}$$

**Figure 3:**
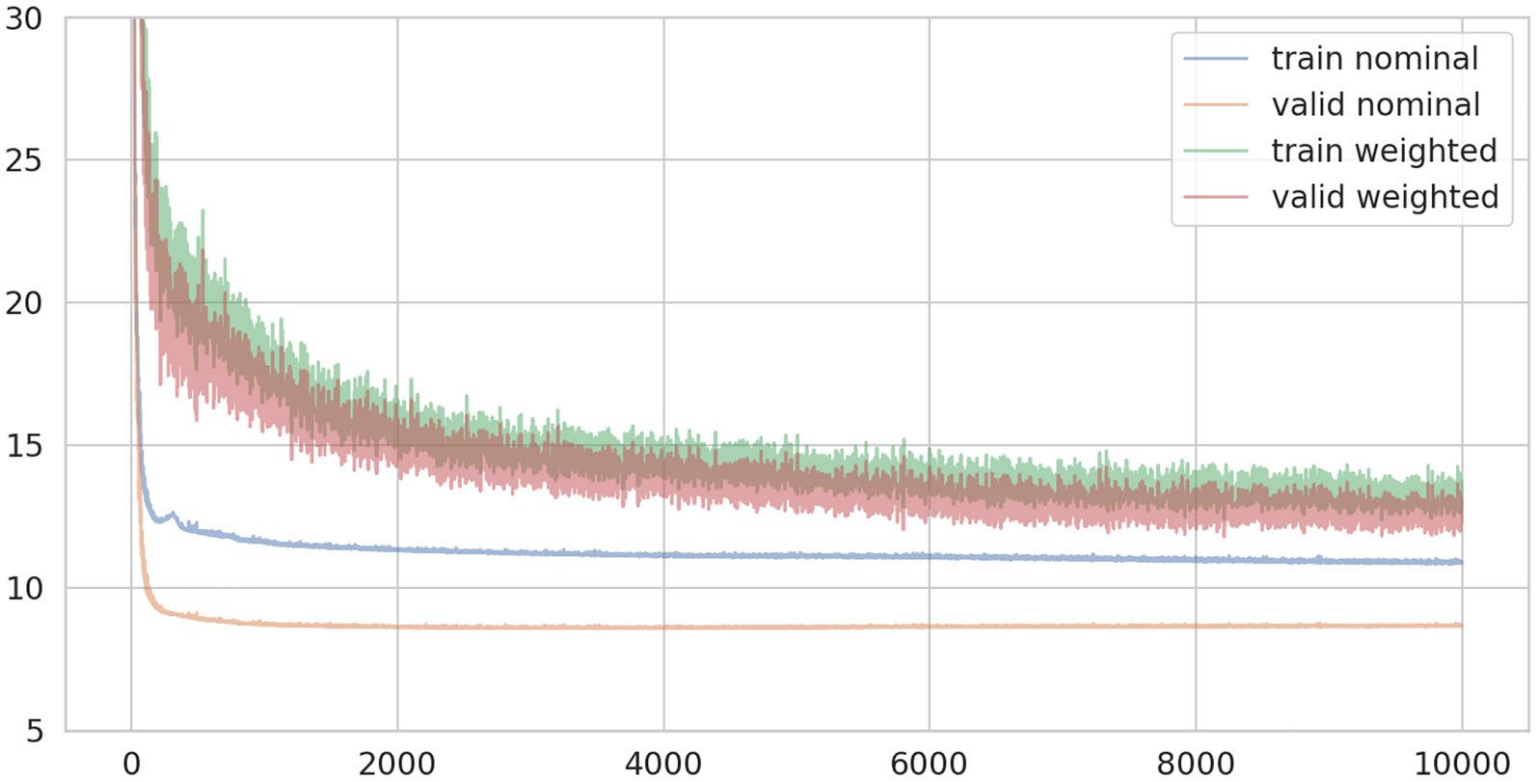
History of the loss function in the 10,000 epochs of the training.

We use the Adam optimizer and a learning rate of $10^{-5}$. During the training, back-propagation is applied after computing the loss on samples extracted from the dataset in batches. The procedure is repeated an arbitrary number of times. Statistics are collected after iterating back-propagation on as many samples as the number of elements in the training dataset: this is called an epoch. The training ends once the loss function stops decreasing on the validation dataset. We used batches of size 256 and stopped training after 10,000 epochs. Examples of the loss function behaviors are presented in Fig. 3.

The code with the implementation of the network architecture and the procedure to generate the training, validation, and test datasets are available as a Python notebook in the public GitLab repository gitlab.fbk.eu/dsip/dsip_physics/dsip_ph_space/Dstw.

### Baseline model and evaluation metric

A typical baseline forecast method for time series is the persistent model. The assumption at the base of this approach is that nothing changes between the last known value and all the future points:

2 $$\begin{aligned} Dst(t + n) = Dst(t),\quad n\in \mathbb {N}. \end{aligned}$$

It is expected that the predictive power of this model will decrease with the increase of the forecast horizon; on the contrary, in the short term, assuming persistence is often a good approximation of the actual trend.

Different metrics can be considered to highlight and study models’ features and compare their predictive power. However, the focus of this work is the importance of how the training data are selected and used. This is appreciable even considering only the most common of these metrics, the Root Mean Squared Error (RMSE), defined as:

3 $$\begin{aligned} \text {RMSE}=\sqrt{\frac{\sum _{i=1}^N \left( y_{pred_i}-y_{true}\right) ^2}{N}}. \end{aligned}$$

## Results

### Dst prediction

Before discussing the importance of processing the data in the training procedure and analyzing the performances obtained with the different approaches, we compared our results with similar *state-of-the-art* calculations available in the literature that uses other networks on similar data.

**Figure 4:**
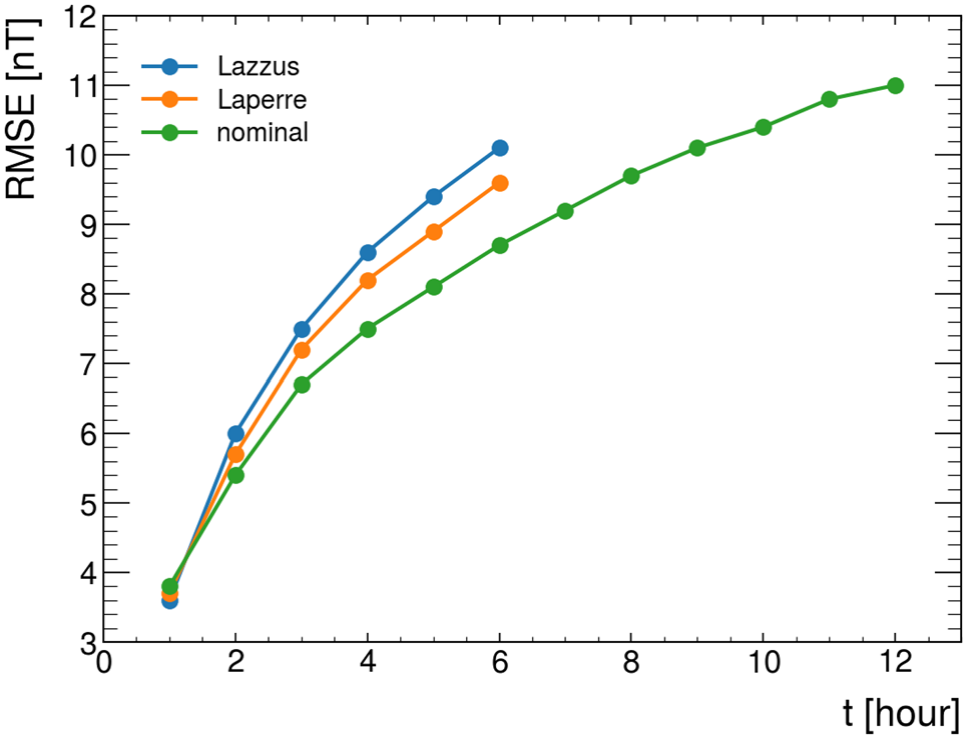
Comparison between the RMSE as a function of the forecast horizon obtained with the nominal neural network presented in this work and two state-of-the-art solutions.

For such a comparison, we consider both^25^, and^26^. In the first, a similar way of splitting the data is used, and their test set has a consistent overlap with ours. The second uses a DL network with similarities with ours but a different approach for the splitting. A comparison of the performances in this last case is more difficult. Having this in mind, Table 1 and Fig. 4 show how our network outperforms the other two approaches.

**Table 1:** Comparison between the predictive power in terms of Root Mean Square Error [nT] as a function of the forecast horizon obtained with the nominal neural network presented in this work and two state-of-the-art solutions.

	Lazzus ^25^	Laperre ^26^	nom
t + 1h	**3.6**	3.7	3.8
t + 2h	6.0	5.7	**5.4**
t + 3h	7.5	7.2	**6.7**
t + 4h	8.6	8.2	**7.5**
t + 5h	9.4	8.9	**8.1**
t + 6h	10.1	9.6	**8.7**
t + 7h	–	–	**9.2**
t + 8h	–	–	**9.7**
t + 9h	–	–	**10.1**
t + 10h	–	–	**10.4**
t + 11h	–	–	**10.8**
t + 12h	–	–	**11.0**

Best results are in [bold].

We also investigated neural networks not using the complete set of input parameters. We are not showing the results here because this type of analysis is lateral to the paper’s main topic. Still, summarizing, we found agreement with^31^ where it was shown that the solar wind electric field and the north-south component of the IMF have a key role in obtaining a good prediction of the Dst index. Furthermore, testing different combinations of input data for the network, we found that the temperature contributes less to the solution’s performance. The SW density, instead, is more significant when combined with other parameters, such as the SW speed.

Established that our network can obtain comparable or even better performances than the analogous solutions available in the literature, we focus now on the importance of training data preparation.

In Table 2 is reported the RMSE obtained until $t+12$ on three different models: the baseline persistence model, the Neural Network (NN) trained with the *nominal* sampling Fig. 1c blue, and the NN trained with the *weighted* sampling Fig. 1c orange. Remember that the difference is not the data used but how they are sampled in each epoch of the training procedure.

**Table 2:** Root Mean Square Error [nT] in train, validation, and test for the three models: persistence, nominal, weighted. The nominal has better performance in the test set, but this is connected with the dataset being highly unbalanced towards Dst values $> -20$ nT.

	Train			Valid			Test		
	Pers	Nom	Weight	Pers	Nom	Weight	Pers	Nom	Weight
t + 1h	5.2	4.7	6.7	4.1	3.8	6.2	4.0	**3.8**	6.0
t + 2h	8.3	6.8	7.8	6.4	5.5	7.1	6.4	**5.4**	6.8
t + 3h	10.2	8.3	9.0	8.0	6.8	8.2	8.0	**6.7**	7.9
t + 4h	11.7	9.3	10.0	9.1	7.6	9.2	9.1	**7.5**	8.9
t + 5h	12.9	10.0	10.8	10.0	8.2	10.2	10.0	**8.1**	9.9
t + 6h	13.9	10.7	11.9	10.7	8.6	11.4	10.8	**8.7**	11.0
t + 7h	14.9	11.4	13.2	11.5	9.1	12.6	11.5	**9.2**	12.3
t + 8h	15.7	12.0	14.7	12.1	9.5	14.1	12.2	**9.7**	13.9
t + 9h	16.4	12.6	16.1	12.7	9.9	15.5	12.8	**10.1**	15.5
t + 10h	17.0	13.1	17.3	13.2	10.3	16.8	13.2	**10.4**	16.7
t + 11h	17.6	13.6	18.1	13.6	10.7	17.4	13.6	**10.8**	17.4
t + 12h	18.1	14.1	18.4	14.0	10.9	17.4	14.0	**11.0**	17.6

Best results are in [bold].

As a side remark, it is worth noting how close are the performances obtained in validation and test. This result is expected given the similar distribution of Dst values for the two datasets (Fig. 1b).

Moving to the comparison of the three models, the RMSE obtained looks smaller for the *nominal* method, and the persistence approach shows better results than the *weighted* model, at least in validation and test. However, is this the correct way to determine the effectiveness of the models to see storms coming? It is crucial to remember that “in production” we are interested in knowing in advance when a storm starts and not predicting quiet periods.

In the validation and test datasets, most of the samples refer to low geomagnetic activity. So if our method is good at predicting those events, the RMSE will be small even if the error is significant in the much more relevant and rarer cases of storms. But this is not the aim of the project.

Therefore, it is crucial to understand how the models perform in different classes of geomagnetic activities. We consider here four classes:

- low $$Dst > -20$$ nT
- medium $-20$ nT$< Dst <-50$ nT
- high $-50$ nT$<Dst<-100$ nT
- intense $$Dst<-100$$ nT

To increase the statistics on low Dst events and since the performances are comparable between validation and test set, in the following, we present results obtained by merging the validation and test datasets. Table 3 shows the RMSE for the three models in the four classes. For low geomagnetic activity ($Dst > -20\ {\text {nT}}$ and $-20\ {\text {nT}}< Dst < -50\ {\text {nT}}$) the *nominal* model obtained the best previsions, and this is expected from what discussed before. On the other hand, the *weighted* model performs better than *nominal* during high geomagnetic conditions ($-20\ {\text {nT}}>Dst>-100\ {\text {nT}}$ and $Dst<-100 \ {\text {nT}}$). This behavior confirms the importance of the re-weighting procedure: The possibility for the network to see storm examples more often during the training procedure considerably improves the ability in its prediction.

**Table 3:** Same as Table  2 but exploding the results in the four different classes of geomagnetic activities. The weighted model perform better than the other two in high and intense geomagnetic conditions.

	Dst$>-20\ {\text {nT}}$			$-20\ {\text {nT}}> Dst > -50\ {\text {nT}}$			$-50\ {\text {nT}}> Dst > -100\ {\text {nT}}$			Dst$< -100\ {\text {nT}}$		
	Pers	Nom	Weight	Pers	Nom	Weight	Pers	Nom	Weight	Pers	Full	Weight
t + 1h	**3.4**	**3.4**	5.8	5.6	**4.1**	6.2	10.0	**9.9**	11.8	**17.0**	18.2	23.6
t + 2h	5.2	**4.7**	6.6	8.8	**6.7**	7.8	17.5	14.5	**13.8**	31.4	28.6	**27.9**
t + 3h	6.3	**5.6**	7.5	11.0	**8.5**	9.1	22.9	18.6	**16.8**	43.2	40.0	**35.0**
t + 4h	7.0	**6.1**	8.5	12.5	**9.7**	10.4	27.4	21.5	**18.4**	54.1	49.8	**43.2**
t + 5h	7.5	**6.4**	9.2	13.7	**10.5**	11.7	31.0	24.4	**20.9**	63.8	56.8	**46.6**
t + 6h	7.9	**6.6**	10.2	14.9	**11.1**	13.0	34.3	26.9	**22.6**	73.2	63.5	**50.4**
t + 7h	8.2	**6.7**	11.2	15.9	**11.7**	14.7	37.6	29.7	**24.1**	81.4	68.3	**53.8**
t + 8h	8.5	**6.9**	12.7	16.8	**12.4**	16.3	40.5	32.1	**25.5**	87.9	73.0	**56.8**
t + 9h	8.8	**7.0**	14.0	17.6	**12.9**	17.7	42.9	34.3	**27.4**	93.4	77.3	**60.1**
t + 10h	9.0	**7.1**	15.3	18.2	**13.3**	18.7	44.9	36.1	**29.0**	97.4	82.4	**64.6**
t + 11h	9.2	**7.2**	15.9	18.9	**13.7**	19.4	46.7	37.8	**30.6**	100.7	86.7	**69.9**
t + 12h	9.4	**7.3**	15.9	19.5	**14.0**	19.9	48.1	39.3	**31.8**	103.4	90.4	**73.8**

Best results are in [bold].

### Dynamic time warping analysis

In^26^ Dynamic Time Warping (DTW) has been suggested as a method to check the level of persistence present in models based on Neural Networks. DTW is a method to estimate the similarity between time series. What characterizes DTW is that the measure takes care of shifts and stretches in time of the series. Given two time series of different lengths *n* and *m*, in the DTW algorithms:

- A grid nxm is made.
- Eeach point (*i*, *j*) of the grid is filled with the distance between the *i*-th element of the first series and *j*-th of the second. The metric can be freely chosen and we use the Euclidean distance.
- The warping path *P* is build that minimizes
  4 $$\begin{aligned} DTW=\min \left( \sqrt{\sum _{i=1}^Kp_i}\right) \end{aligned}$$
  where $p_k$ is a point of the grid and the length *K* of the path $P=[p_1,p_2,\ldots ,p_K]]$ is $\max (m,n)\le K\le m+n-1$

Conditions that must be satisfied by path P are that: the beginning and end of the series are matched; every point is mapped at least with one of the other series; the elements are ordered in time.

Using this method, we can check immediately if the original time series and the prediction differ only for a constant shift, as in the case of the persistence model. Following^26^ we test DTW for the *nominal* and *weighted* solution.

From the results presented in Table 4 emerges that a certain degree of persistence is present in the *nominal* case. Indeed, the larger values of the coefficients in each row are for the corresponding time-shift column. Nevertheless, the values are much smaller in the *weighted* case (Table 5), showing once again the importance of the different sampling of the events during training.

**Table 4:** The row-normalized fractions of the DTW measure for the network trained with the nominal data.

	0 h	1 h	2 h	3 h	4 h	5 h	6 h
t + 1h	**0.37**	**0.37**	0.12	0.07	0.05	0.01	0.01
t + 2h	0.15	0.31	**0.33**	0.13	0.04	0.02	0.02
t + 3h	0.08	0.14	0.25	**0.31**	0.11	0.07	0.04
t + 4h	0.08	0.08	0.15	0.22	**0.26**	0.13	0.08
t + 5h	0.07	0.09	0.05	0.13	0.24	**0.26**	0.15
t + 6h	0.11	0.11	0.07	0.11	0.12	0.22	**0.27**

Best results are in [bold].

**Table 5:** The row-normalized fractions of the DTW measure for the network trained with the reweighted data.

	0 h	1 h	2 h	3 h	4 h	5 h	6 h
t + 1h	**0.34**	0.21	0.14	0.08	0.11	0.08	0.05
t + 2h	0.17	0.19	**0.27**	0.12	0.09	0.07	0.09
t + 3h	0.13	0.13	0.23	**0.17**	0.15	0.12	0.08
t + 4h	**0.18**	0.10	0.15	**0.18**	**0.18**	0.14	0.07
t + 5h	0.19	0.08	0.09	0.12	**0.20**	0.19	0.13
t + 6h	**0.23**	0.07	0.15	0.04	0.11	**0.23**	0.17

Best results are in [bold].

## Discussion

From the perspective of developing a warning system for geomagnetic storms influencing the Earth’s magnetosphere, it is interesting to convert our models into *classification models* which try to predict the Dst-index class. To accomplish this task, we associate the output value of the models to the corresponding Dst class.

In evaluating the performance of these classifiers, the accuracy (ACC) alone can be a misleading measure when facing an unbalanced dataset. For this reason, we also consider the geometric mean (G-Mean), which is more sensitive to errors in underrepresented classes. The Geometric mean is defined as:

5 $$\text {G-Mean} = \sqrt{\text {Sensitivity} * \text {Specificity}},$$

6 $$\begin{aligned}&\text {Sensitivity} = \frac{\text {TP}}{(\text {TP} + \text {FN})} \end{aligned}$$

7 $$\begin{aligned}&\text {Specificity} = \frac{\text {TN}}{(\text {FP} + \text {TN})} \end{aligned}$$

Sensitivity, computed by counting the number of true positive (TP) and false negative (FN) cases, refers to the true positive rate and measures the ability to predict the correct class. Specificity instead focuses on how well the negative class is predicted by looking at the true negative (TN) and false positive (FP) occurrences. Combining these two scores into a single number gives the geometric mean. We indicate as ‘classification score’ the couple (ACC, G).

It can be easily seen from Table 6 that, as expected, at $t+1$ the persistence approach provides better previsions than the weighted model. Such a result changes at both $t+6$ and $t+12$. In fact, the persistence model completely fails in predicting storming periods, while the weighted is able to correctly predict stormy periods at both $t+6$ and $t+12$. Globally for $t+12$, the persistence model has an overall score equal to (0.84, 0.32) while the weighted has an overall score (0.77, 0.39).

**Table 6:** Accuracy and G-Mean classification scores obtained for the models at three different forecast horizons.

	Pers	Nom	Weight
	(ACC, G-Mean)	(ACC, G-Mean)	(ACC, G-Mean)
t + 1h	(0.95, 0.84)	(0.96, 0.76)	(0.92, 0.78)
t + 6h	(0.88, 0.52)	(0.90, 0.41)	(0.85, 0.59)
t + 12h	(0.84, 0.32)	(0.88, 0.16)	(0.77, 0.39)

The difficulty in establishing the best model by looking simultaneously at accuracy and G-Mean shows that we cannot verify the model’s performances by focusing only on global scores for this problem with highly unbalanced datasets and multiclass. Instead, it is more informative to analyze the confusion matrices from where we can extract information about efficiency in each class. This is also considering that we are interested in quantifying how often we could issue a reliable alert using these models: a clear understanding of the goodness in the classes connected with intense storms is crucial.

**Figure 5:**
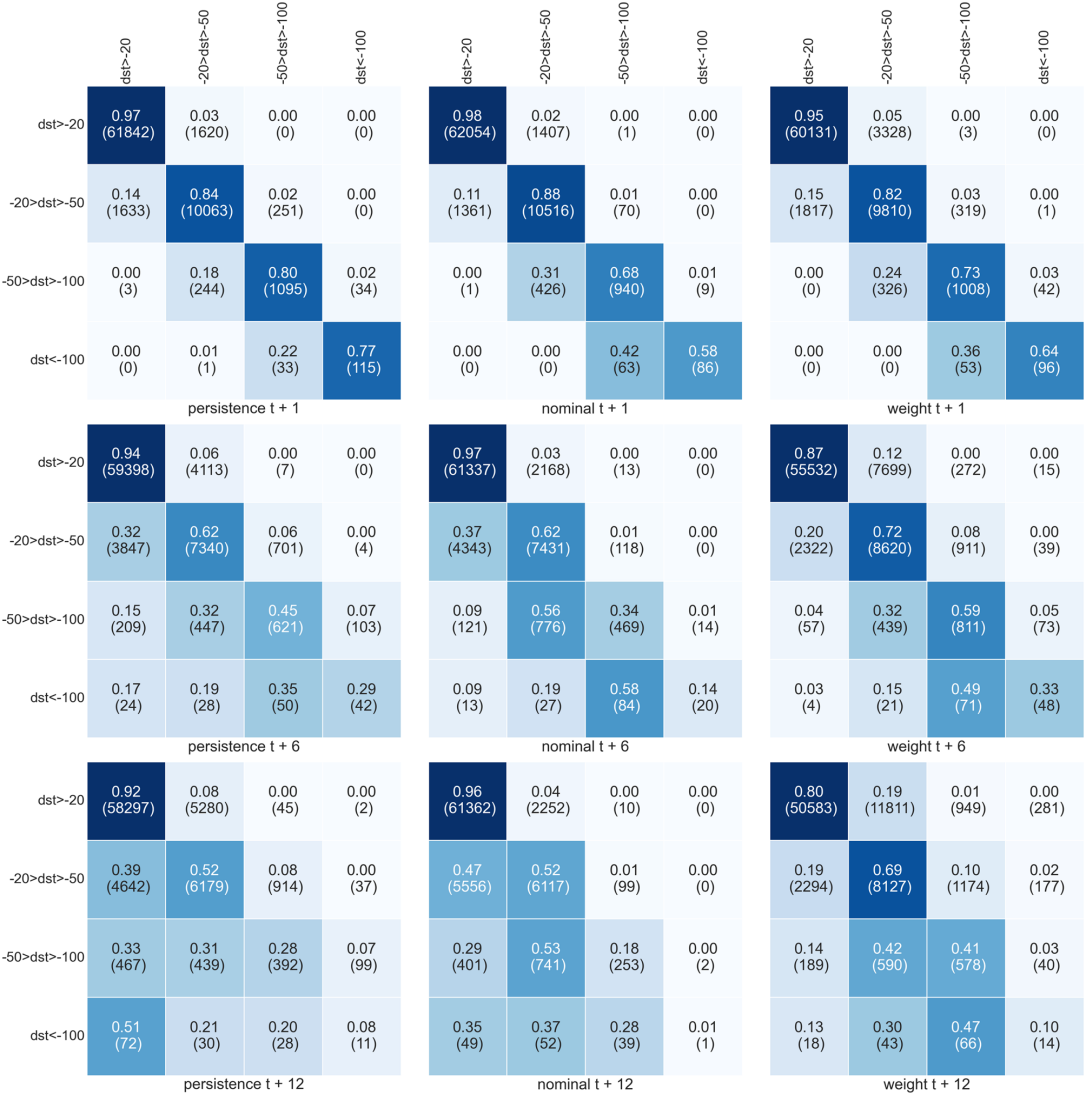
These are the confusion matrices obtained by mapping the output of the three models into the four classes of geomagnetic activity. The color of the heatmaps corresponds to the accuracy of the predictions.

Figure 5 shows the confusion matrices at $t+1$ (upper panels), $t+6$ (middle panels) and $t+12$ (lower panels) for the persistence model on the left, *nominal* model in the center and *weighted* model on the right. The colors, from from light to dark blue, are representative of the percentage of events collected in each class, namely: light blue corresponds to the $0\%$ and dark blue to the $100\%$.

The first row of the figure confirms that the persistence model is good at one-hour forecasting. The situation changes significantly for predictions at $t+6$ and $t+12$. From the confusion matrices, it is immediately clear that the global better score obtained for the persistence model was an artifact of the highly unbalanced dataset where the first class of events is much more frequent but not interesting. Excluding this first class, the performances of the reweigthed model are better in all the other three. Moreover, not only the values on the diagonals are higher (ability to predict the class correctly), but an incorrect prediction is more likely to be associated with a contiguous class than the correct one.

This behavior emerges clearly from the confusion matrices in the bottom row of the figure. Although the reweighted model fails most of the time to predict events in the last class, it can distinguish stormy from quiet periods much better than the persistence or nominal model. On the single last class, the accuracy is only $11\%$ while summing high and intense storms we reach $57\%$, this against $28\%$ of the persistence and $29\%$ of the nominal.

As a final step, we consider the ability of the models to predict storms when the Dst values in the input data are associated only with low or medium activities. The performances in this condition are particularly relevant because predicting a storm coming from data in a quiet period is much more helpful than having the prediction when we are already in the middle of a dramatic event.

**Figure 6:**
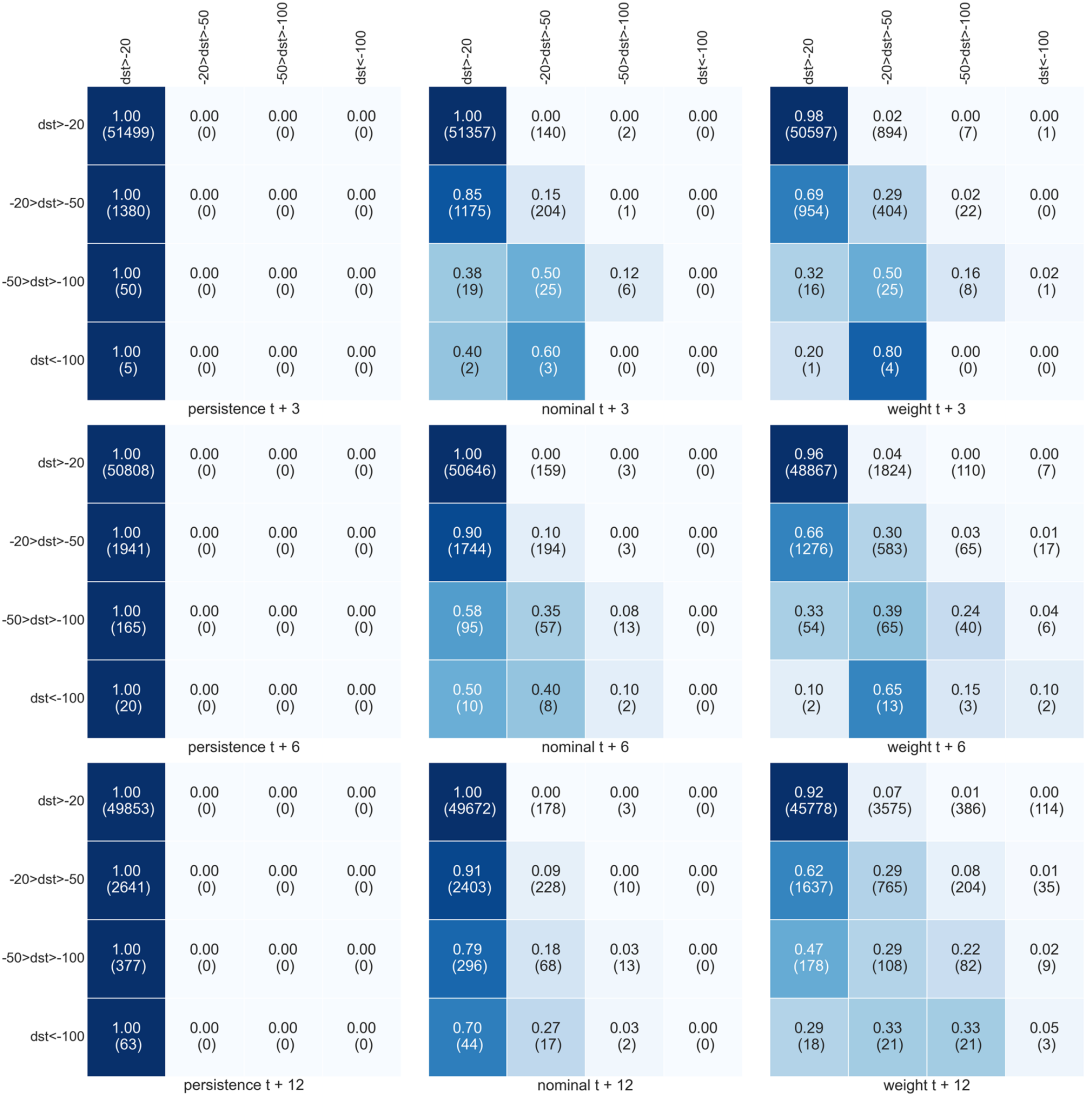
Same as Fig. 5 but only selecting input data where elements have Dst all larger than $-20$.

**Figure 7:**
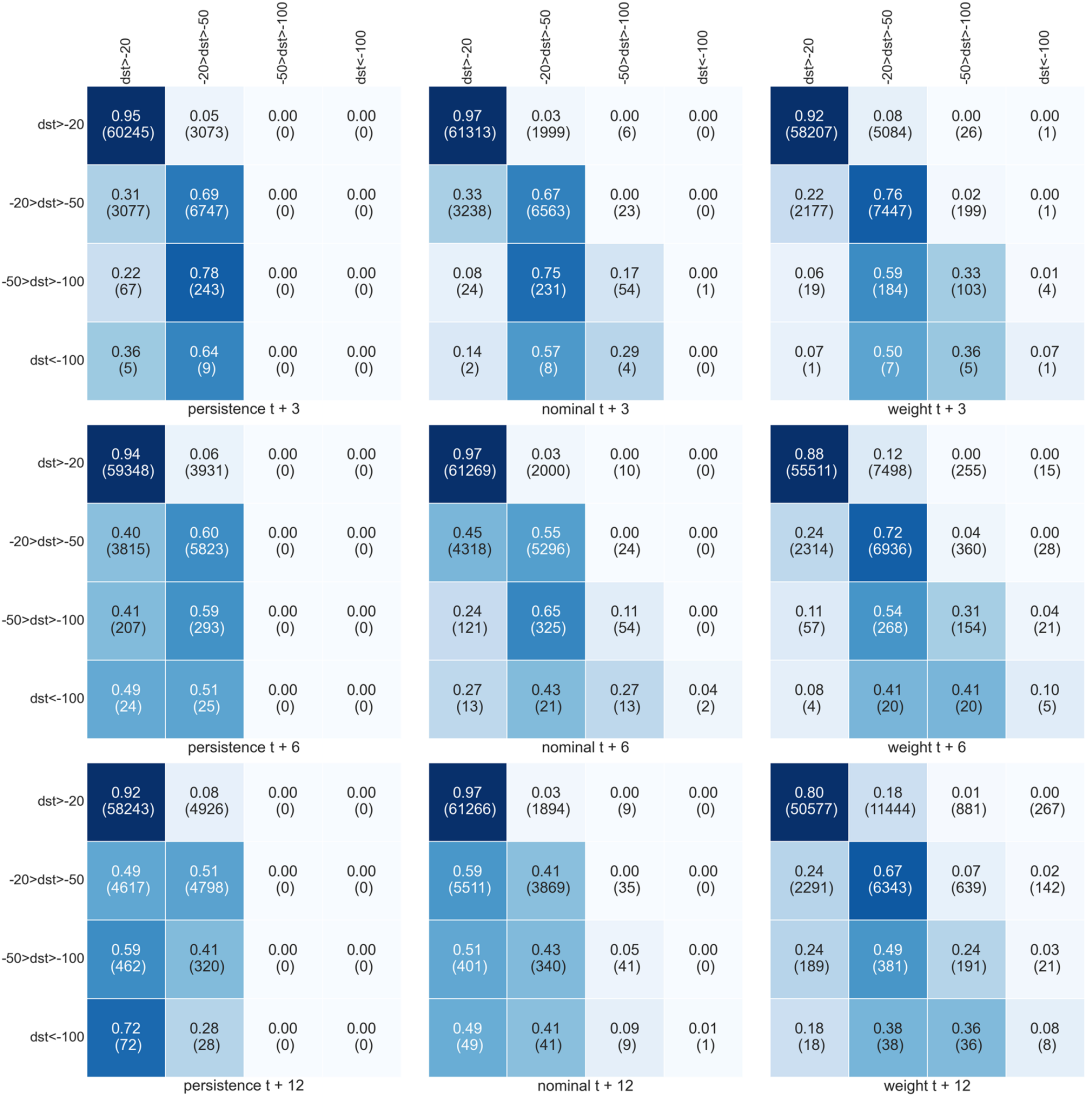
Same as Fig. 6 but here, all the input elements have Dst larger than $-50$.

Figure 6 shows the confusion matrix obtained when all the 12 elements of the input time series have $Dst>-20$ nT. The persistence model fails by definition in all the classes except the first. It is instead remarkable to notice how a not insignificant number of times, the rewighted model can forecast stormy and disturbed geomagnetic periods both at $t+6$ and $t+12$. The same occurs relaxing the constraint permitting $Dst>-50$ nT as input data as shown in Fig. 7).

## Conclusions

In this research, we introduced a detailed data analysis plan for the development and validation of algorithms based on Deep Learning Neural Networks to predict the Dst index during both quiet and disturbed geomagnetic conditions using the interplanetary magnetic field and the solar wind parameters. Our analysis shows that both what we called nominal and weighted models provide better results than the persistent benchmark and other state-of-the-art neural network architectures. The response of the neural networks to training procedures that differ in the preparation of the training dataset was investigated. We strongly demonstrated that the training procedure strictly changes the capability of giving a correct forecast of stormy and disturbed geomagnetic periods. Indeed, the strategy proposed for the training dataset selection and the optimization of the architecture plays a key role in the algorithm’s performance.

## Data Availability

The datasets analysed during the current study are available in the OMNIWeb online repository, http://omniweb.gsfc.nasa.gov/.
